# Automated machine learning for fabric quality prediction: a comparative analysis

**DOI:** 10.7717/peerj-cs.2188

**Published:** 2024-07-23

**Authors:** Ahmet Metin, Turgay Tugay Bilgin

**Affiliations:** Bursa Technical University, Bursa, Turkey

**Keywords:** AutoML, Quality control, Imbalanced data, Hyperparameter optimization, Model interpretability, Feature importance, Textile industry

## Abstract

The enhancement of fabric quality prediction in the textile manufacturing sector is achieved by utilizing information derived from sensors within the Internet of Things (IoT) and Enterprise Resource Planning (ERP) systems linked to sensors embedded in textile machinery. The integration of Industry 4.0 concepts is instrumental in harnessing IoT sensor data, which, in turn, leads to improvements in productivity and reduced lead times in textile manufacturing processes. This study addresses the issue of imbalanced data pertaining to fabric quality within the textile manufacturing industry. It encompasses an evaluation of seven open-source automated machine learning (AutoML) technologies, namely FLAML (Fast Lightweight AutoML), AutoViML (Automatically Build Variant Interpretable ML models), EvalML (Evaluation Machine Learning), AutoGluon, H2OAutoML, PyCaret, and TPOT (Tree-based Pipeline Optimization Tool). The most suitable solutions are chosen for certain circumstances by employing an innovative approach that finds a compromise among computational efficiency and forecast accuracy. The results reveal that EvalML emerges as the top-performing AutoML model for a predetermined objective function, particularly excelling in terms of mean absolute error (MAE). On the other hand, even with longer inference periods, AutoGluon performs better than other methods in measures like mean absolute percentage error (MAPE), root mean squared error (RMSE), and r-squared. Additionally, the study explores the feature importance rankings provided by each AutoML model, shedding light on the attributes that significantly influence predictive outcomes. Notably, sin/cos encoding is found to be particularly effective in characterizing categorical variables with a large number of unique values. This study includes useful information about the application of AutoML in the textile industry and provides a roadmap for employing Industry 4.0 technologies to enhance fabric quality prediction. The research highlights the importance of striking a balance between predictive accuracy and computational efficiency, emphasizes the significance of feature importance for model interpretability, and lays the groundwork for future investigations in this field.

## Introduction

According to the large-scale study carried out by Wang et al. (2016), careful IT integration serves as a revolutionary driver in a variety of industry sectors. Large-scale datasets included in big data systems, cloud computing infrastructure, advanced machine learning (ML) techniques, and IoT are all integral and revolutionary elements of this thorough and harmonious integration process. This development is ascribed to the idea of Industry 4.0, which represents a fundamental change in industrial procedures and practices and has gained broad recognition. Even though it possesses one of the most intricate industrial chains and operates with a high degree of automation, the textile production sector is seen as being in the early stages of incorporating Industry 4.0 technologies. The emergence of the Industry 4.0 revolution brings considerable potential for the textile industry, offering the prospect of improving production efficiency, cutting costs, and simplifying quality control procedures.Textile manufacturing firms are under constant pressure in the current competitive market from rising consumer expectations for customized items that exhibit a higher quality while also having a reduced production cost. Following the orders that are received and the designs that have been made, production follows the planning process. If the final product fulfills the requirements of the quality control process, it is delivered. However, the production procedure and the final product must be routinely examined by the quality control process, and any errors must be fixed and new plans must be made. To meet the objectives of quicker delivery times and better textile quality in this situation, adopting intelligent integration solutions is the best course of action for the entire supply chain. The typical method, as noted by Tavana, Hajipour & Oveisi (2020), is to collect data from the ERP system and then transfer the data to the cloud platform for statistical analysis. The information gathered relates to the characteristics of the fiber, the process parameters, the yarn, the requirements for the loom, and the machine feature taken from the firm ERP. Raw materials go through a number of processes where fibers and yarns are joined, and the combination of these yarns goes through a number of textile processes, eventually generating a fabric as its end product. In numerous stages of this production process, automation technologies might be used.

In the loom’s operation, the insertion of weft threads into warps is a fundamental step. This process involves introducing the weft, threading the needle *via* the fabric’s shedding, and utilizing the reed pulses to move the inserted thread across the fabric that has already been created, as outlined in Azevedo et al. (2022). The warp yarns may break as a result of this procedure. The pressure that the yarn is put under during this process will show where the basic material is weak. As a result, while under tension, a yarn with thin spots would usually break, as opposed to other spots on the yarn. The amount of friction between the threads will rise as the process speed rises. Because of this, the procedure will get more tense, which will result in more breaks. In the production of densely threaded fabric, it is worth noting that thicker areas and neps can occasionally contribute to increased friction between the threads. The three primary problems related to yarns during this process are weft tears, warp ruptures, and yarn explosions. Whenever any of these difficulties arise, the machine must be stopped to allow the operator to reconnect the broken yarns before production may resume. Industrial output interruptions are a critical concern in manufacturing because they have an immediate influence on productivity, effectiveness, and profitability. Reduced downtime leads to higher machine availability, which improves production capacity, shortens delivery processing times, and elevates customer happiness. Additionally, the causes of loom downtime, as well as the length and amount of each downtime, generate a lot of data and require a lot of storage.

The efficiency of the weaving process is contingent upon a multitude of variables encompassing the technical condition of the weaving machinery, the quality of the utilized yarns, the proficiency of the workforce, and prevailing environmental conditions. Each of these factors collectively contributes to optimizing the utilization of the weaving apparatus, ultimately ensuring the production of high-quality woven fabric. Contemporary weaving machines boast advanced technical features that facilitate an enhanced exploitation index, accompanied by elevated operational speeds and continuous monitoring of critical technical parameters. Notably, the frequency of warp and weft thread breakages per meter of fabric or per unit of time significantly influences the overall operational efficiency of the weaving machinery. Given the multifaceted nature of this phenomenon, it becomes imperative to employ a diverse array of methodologies for monitoring and assessment, considering the multifarious variables affecting thread breakages. In the pursuit of predicting and evaluating the quality of the weaving process, particular emphasis is placed on monitoring the incidence of thread breakages.

Based on Kraußet al. (2019), benefits from applications like ML-based product quality prediction include reducing repair costs and shortening manufacturing lead times, as well as enhancing client relationships and having a better understanding of the root causes of issues. However, applying ML effectively is not a simple task. Data scientists must prepare the data (for instance, by encoding categorical characteristics), choose an ML method, and adjust its hyperparameters in order to produce meaningful machine learning models from the data. This requires in-depth knowledge, such as understanding which hyperparameters to adjust and how. Even with this knowledge, it still requires a lot of work to make optimal decisions because they are unique to each data set and occasionally depend on one another, claim Van Rijn & Hutter (2018) and Probst, Boulesteix & Bischl (2019). The concept of AutoML, which involves automating the tasks that must be completed inside ML projects, offers a way to circumvent this resource shortage. By automatically generating wise judgments, AutoML lets individuals save precious resources, such as time, money, and human resources. Since effective machine learning algorithms and their hyperparameter settings are critical to the effectiveness of data learning, amalgamated algorithm selection and hyperparameter tuning become an important task in general AutoML systems as well as information processing pipelines. While feature engineering and data pre-processing are important factors in the results of information analysis, automating these processes is still difficult and frequently requires human involvement.

The goal of this study is to assist production planning using data acquired by IoT sensors and ERP systems from textile machines, as well as to produce a new quality forecast for each product based on imbalanced fabric quality data. In this approach, fewer people are needed for the quality control process. In this study, AutoML is used to streamline the ML training part and, as a consequence, shorten the data maintenance clutching operation process, as opposed to the traditional ML design by experts technique. It also stresses selecting the most suitable supervised ML method and optimizing the hyperparameters associated with it. Seven contemporary open-source AutoML technologies are taken into account in the comparison study: FLAML, AutoViML, EvalML, AutoGluon, H2OAutoML, PyCaret, and TPOT.

## Related Work

The literature’s present state as well as prior studies on AI-based quality control systems and textile forecast time improvement are covered in this part. The information technology tools available today effectively evaluate data and turn it into knowledge. Data mining (DM) is a method for identifying previously undiscovered but possibly relevant patterns in unprocessed data. Statistics, database technology, machine learning, artificial intelligence (AI), and visualization are all used in the multidisciplinary discipline of DM.

DM makes extensive use of ML techniques. ML can offer automatic learning ways to find patterns in data, and large datasets can be utilized to generalize knowledge. The textile industry generates and stores a large amount of data as well. Parameters for the product’s quality, machine configurations, and raw materials are covered in these elements. The textile business places a lot of emphasis on quality specification management. DM can be used to identify useful criteria for product quality. However, conventional human control might result in poor judgment, higher expenses, and sluggish productivity. In predicting yarn quality, Mozafary & Payvandy (2014) employed data-mining approaches such as clustering and artificial neural networks (ANN). The findings indicated that the DM technique exhibited superior performance compared to ANN. Additionally, Yildirim, Birant & Alpyildiz (2018) conducted a comprehensive exploration of DM methods specifically designed for textile applications, providing an overview of various experimental endeavors documented in the literature. The review underscores the utility of clustering and classification algorithms in addressing textile industry challenges. Importantly, the research highlights a prevalent preference for classification techniques over clustering methods within the textile sector. In a different study, Bo (2010) utilized an ANN model to investigate the prediction of warp rupture rate based on the sizing yarn characteristics parameter. The outcomes of this study, supported by subsequent research, provide evidence for the effectiveness of ANN-based models in predicting quality within the textile industry. ANN finds another application in predicting the breaking elongation of ring-spun yarn, a crucial parameter that significantly influences the production and utilization of woven and knitted textiles. In their research, Mwasiagi, Huang & Wang (2008) investigates the performance of ANN when adjusting design aspects to forecast the breaking stretch of cotton ring-spun thread. The study identifies six key input variables crucial for ANN prediction, underscoring their relative significance in the predictive model.

There are supervised machine learning algorithms that typically result in data-driven models with great projected accuracy, despite the fact that the resulting models are difficult for humans to understand. As a consequence, such models tend to be called “black boxes” because of their intrinsic complexity and the difficulty in grasping the fundamental operations of the algorithms. Enhancing interpretability in black-box machine learning models can be effectively achieved through two primary approaches: rule extraction and visualization techniques. The studies conducted by Cortez & Embrechts (2013); Cortez & Embrechts (2011), emphasize the importance of explanation capacity in their investigations. This element refers to the capacity to extract knowledge from machine learning models in a human-comprehensible format. Additionally, it presents a unique visualization tool built on sensitivity analysis (SA), an essential approach for determining the consequences of changing an input value on a model’s output. Mainly, sensitivity analysis was used as an attribute decision approach, identifying the least significant feature throughout each phase of a backward selection process that should be discarded. However, like the aforementioned variable effect characteristic (VEC) curve, SA may be used to describe the model and unlock the “black box.”

A quality machine learning lifecycle is based on the proper tool, the right human collaboration, and the collection of sound data when it comes to the process of extracting value from data. It is a serious problem that the degree to which this sector achieves the required success depends on the availability of trained personnel in a world where demand for qualified employees in the field of machine learning is rising. This issue was resolved by the creation of the AutoML idea by Feurer et al. (2015). According to Özdemir & Örslü (2019) no matter the real-world obstacle, it is desirable to approach the machine learning process of the problem, including the data, as an optimization problem using an iterative or linear pipeline architecture. The hyperparameters used to tailor the majority of machine learning algorithms must be carefully chosen because their values frequently have a considerable impact on performance. Various autonomous hyperparameter optimization (HPO) algorithms may be used to discover high-performing hyperparameter combinations, substituting the laborious and unreliable human experimentation approach. Following a broad introduction to HPO, Bischl et al. (2023) delves into significant HPO methodologies. Simple methods like grid or random search are among them, as are more complex ones like Evolution strategies, Hyperband, Bayesian optimization. Regarding crucial decisions to be made when doing HPO, this article offers helpful recommendations. In similar research, Ferreira et al. (2021) compared eight modern, freely available AutoML applications. The goal was to compare the differences between these tools and use a lexicographic method to determine the best AutoML tools for each case. The selection criteria included prioritizing the task’s highest mean estimation score, followed by its least computing effort. Wang et al. (2022) proposes an innovative framework called ML-aided MOO-MCDM (Machine Learning-aided Multi-Objective Optimization and Multi-Critical Decision Making) to boost data-driven research. The framework has seven distinct processes, commencing with the establishment of application-specific objectives, constraints, and machine learning models. The process consisted of choosing and developing machine learning models, fine-tuning them with a global or advanced optimization algorithm (like particle swarm optimization), creating a multi-objective optimization problem for NSGA-II to solve, and then conducting a methodical, multi-criteria decision-making analysis to identify a single optimal strategy of action. In order to increase overall performance, particularly in minority classes, and to strengthen the model’s capacity to handle unbalanced datasets, oversampling approaches can be incorporated into the pipeline. The features of the dataset and the particular objectives of the machine learning challenge determine which oversampling strategy is best. In order to manage three different datasets of customer churn characteristics and address the dataset with imbalanced class distribution concerns utilized in the churn recognition problem, Fatima et al. (2024) employ an AutoML oversampling approach. In order to enhance performance, the study makes advantage of the power of oversampling techniques such synthetic minority oversampling with encoded nominal and continuous features (SMOTE-ENC) and synthetic minority oversampling technique (SMOTENC) for nominal and continuous features.In order to enhance performance, the study makes advantage of the power of oversampling techniques such synthetic minority oversampling with encoded nominal and continuous features and synthetic minority oversampling technique for nominal and continuous features. According to AutoML data, the suggested approach performs better than conventional approaches with SMOTE, particularly Random Forest (RF) with SMOTE-NC.

The Cross-Industry Standard Process for Data Mining (CRISP-DM) methodology has evolved as a standardized framework to aid in the execution of real-world machine learning projects, addressing the escalating interest in DM, as elaborated by Wirth & Hipp (2000). Achieving success in the CRISP-DM process necessitates collaborative interaction between experts in both the business domain and DM and machine learning. Multiple iterations are often essential to refine and enhance the model as insights are gained and the understanding of the problem deepens. The use of CRISP-DM techniques in the textile industry is more recent, and it mostly addresses categorization issues like defect detection and yarn quality estimation. Ribeiro et al. (2020a) used the CRISP-DM approach to forecast the rip strength of cloth in both the warp and weft orientations. Three CRISP-DM phases were used for regression tasks, examining data preprocessing techniques like outlier removal and additional inputs. The best weft test results and warp tear strength predictions were obtained during the third and second iterations, respectively. The manufacturing industry relies on assessing production time to optimize plans and meet client deadlines. In their investigation, Sousa et al. (2022) utilized AutoML in conjunction with the CRISP-DM technique to estimate the duration of production. They leveraged various phases of CRISP-DM to assess the time required for completing a manufacturing demand. Additionally, they employed four new open-source AutoML tools to automate method selection and hyperparameter adjustments throughout the analysis stages of CRISP-DM. In a comparable research endeavor, Ribeiro et al. (2020b) explored three raw characterization techniques for textile development and completing operations, utilizing data from the textile industry in two cycles of the CRISP-DM methodology. The primary aim was to identify the most suitable regression model for the specific context. The results demonstrate its potential for cost and process savings by showing that it has the potential to reduce manufacturing costs and the volume of fabric fabrication activities. In their research, Azevedo et al. (2022) provide a machine learning approach for predicting fabric defects, which are frequently linked with production downtime delays. The researchers use the AutoML technique, which allows them to compare multiple ML algorithms. This strategy seeks to increase the autonomy and effectiveness of their production scheduling and oversight while lessening the modeling strain. Furthermore, the selected H2OAutoML model tool underwent a Sensitivity Analysis Explainable Artificial Intelligence (SA XAI) technique. This approach was employed to showcase its potential in acquiring crucial explanatory knowledge within the researched textile field.

## AutoML Methodology

The success of a machine learning lifecycle depends not only on the appropriate technology but also on the correct human involvement in the task of obtaining insights from data. Furthermore, it is contingent on the availability of high-quality data. Despite the abundance of innovative and effective instruments made possible by technical advancements for this cycle, a significant bottleneck is caused by the dearth of qualified individuals. The critical outcomes of human error in the machine learning process, the development of better or worse models based on experience, and the occurrence of bias (bias, deviation, or error in the model due to prejudices) have all accelerated the process of finding a solution, particularly in the recent period. The primary solution for this procedure is AutoML.

**Figure 1:**
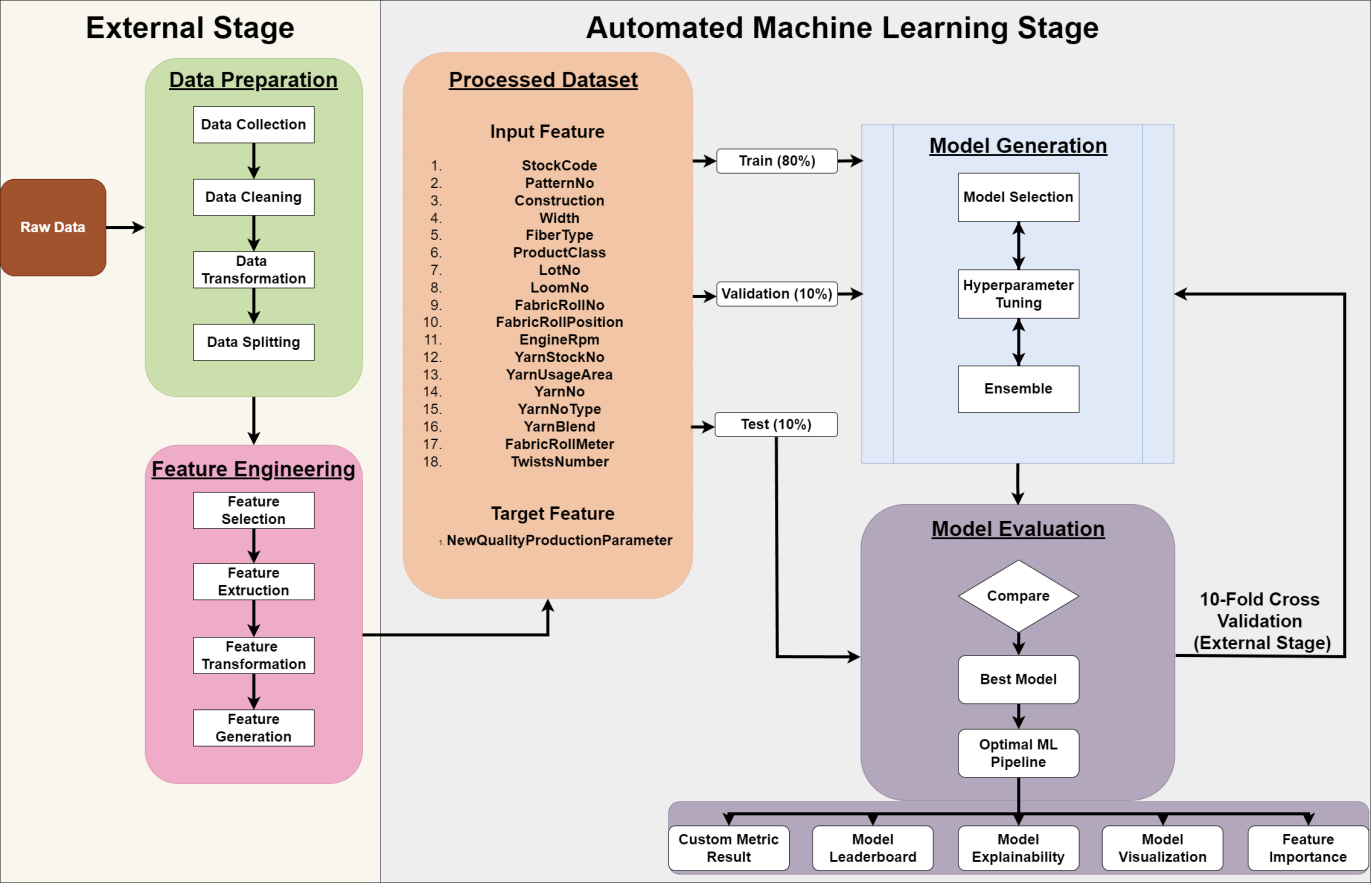
AutoML workflow diagram.

The nomenclature ‘AutoML’, denoting automated machine learning, pertains to the comprehensive automation of machine learning model utilization for the resolution of real-world challenges. By making machine learning more approachable, AutoML assists in reducing its perception as a black box. Because it produces answers that are comprehensible and repeatable, AutoML holds out a lot of promise for solving AI problems in regulated businesses. Without prior knowledge of model development, hyperparameter tweaking, or feature engineering, it promises huge productivity advantages to people and organizations who may lack the competence to create reliable machine learning models. It also makes it possible for less technically skilled personnel to build and use dependable machine learning pipelines, providing a high-performing model while shortening training and development times and enabling machine learning in sectors that have not previously used machine learning models. Simultaneously, rather than endeavoring to supplant data scientists, this technological advancement aspires to alleviate them from the burdensome demands of routine tasks. Despite the fact that feature processing is advertised as being automatic, some AutoML models, like TPOT, do not support this procedure. As highlighted by Feurer et al. (2015), contemporary AutoML tools still grapple with challenges related to insufficient data and missing values, often leading to issues such as crashing. Truong et al. (2019) indicate that there is a noticeable increase in variability in the AutoML tools’ performance when there are more category characteristics present. This is due to the fact that various tools code categorical values in different ways, which produces varying levels of performance. Because of this, AutoML processes like data preparation and feature engineering are not covered in this article. Any potential faults are removed from the start by performing all of these tasks by hand. Thus, both repetitive processes were avoided, and it was possible to compare different AutoML models. Figure 1 depicts each of these steps in detail.

In the majority of instances, the attainment of optimal analytical outcomes and the provision of dependable assessments through the utilization of a randomly selected machine learning model possessing a default architecture or hyperparameter configuration prove to be an insurmountable challenge. To guarantee a comparable outcome, Ferreira et al. chose not to apply the AutoML tools’ hyperparameters in their study. Diverging from the methodology of the Ferreira et al. study, one of the primary motivations for conducting this investigation lies in the inherent similarity of machine learning models utilized across various AutoML platforms, as evidenced in Table 1. When the table is examined, it is easily seen that most AutoML programs employ similar algorithms, with the exception of those that are modified for time series analysis and come within the scope of fundamental linear-based algorithms. It would be more meaningful to compare HPO approaches as opposed to machine learning models, which are constant across platforms, in order to make meaningful comparisons.

**Table 1:** Comparative table of machine learning models used in AutoML tools.

**Machine learning model**	**FLAML**	**AutoViML**	**H2O**	**EvalML**	**TPOT**	**PyCaret**	**AutoGluon **
Ridge Regressor		✓			✓	✓	
Bayesian Ridge Regressor						✓	
Lasso Regressor		✓			✓	✓	
Baseline Regressor				✓			
Least Angle Regression						✓	
ElasticNet				✓	✓	✓	
Exponentia lSmoothing Regressor				✓			
Lasso Least Angle Regression						✓	
Huber Regressor						✓	
Passive Aggressive Regressor						✓	
Dummy Regressor						✓	
Linear Regression		✓		✓		✓	✓
Generalized Linear Model			✓				
Decision Tree Regressor		✓		✓	✓	✓	
Linear Support Vector Regressor		✓		✓	✓		
ExtraTrees Regressor	✓	✓		✓	✓	✓	
Random Forest Regressor	✓	✓		✓	✓	✓	✓
Distributed Random Forest			✓				
Extremely Randomized Trees			✓				✓
CatBoost Regressor	✓			✓		✓	✓
AdaBoost Regressor		✓			✓	✓	
Light Gradient Boosting Machine	✓			✓		✓	✓
K Nearest Neighbors Regressor					✓	✓	✓
XGBoost	✓		✓	✓	✓	✓	✓
Stochastic Gradient Descent Regressor		✓			✓		
Gradient Boosting Machine			✓		✓	✓	
Bagging Regressor		✓	✓	✓			
Fully Connected Deep Neural Network			✓				✓
ARIMA Regressor				✓			
Prophet Regressor				✓			
TimeSeriesBaseline Estimator				✓			
VowpalWabbit Regressor				✓			
Multimodal Predictor							✓

Every machine learning study has hyperparameters, and the goal of the analysis process is to use these hyperparameters to maximize the machine’s performance. Another significant issue in the machine learning process is the essential task known as HPO. It fulfills multiple crucial purposes, making it a crucial part of the machine learning process. Firstly, it is instrumental in the exploration and identification of hyperparameter configurations that exhibit superior performance across a diverse spectrum of datasets. Secondly, HPO facilitates the design and construction of versatile machine learning pipelines tailored to specific application domains, thereby enhancing their adaptability and efficacy. Lastly, it contributes significantly to the refinement and expansion of default hyperparameter configurations prevalent in widely used machine learning libraries and packages. The final model, whether the best individual model or a collection of models, is selected.

To speed up model discovery and hyperparameter efficiency, a space of parameters may be pruned using a variety of strategies, minimizing the time required for optimization. The tools first try to quickly identify an initial parameter set. Some AutoML techniques use preprocessed “meta-features” from trained datasets, each with a known “meta-learner.” The closest “meta-learners” are used as the first model after locating a similar dataset using “meta-features” given a target dataset. The second strategy is to pick a machine learning model and leverage the connection between HPO and the model that was chosen. Fixing the maximum runtime that the tools are permitted to use while looking for the optimal model is the third strategy. Additionally, runtime budgets are essential in an AutoML benchmark because the majority of AutoML frameworks are built to continue operating until a specified time budget is reached. By removing the expense of numerous trial-and-error procedures based on the individual’s experience, AutoML seeks to address the HPO issue and enable the machine learning process to be employed in a seamless workflow. There are some important approaches frequently used in the literature:

• Grid search (GS) and random search (RS): When exploring the hyperparameter space for the first time, these fundamental techniques are frequently used. In a grid search, a collection of hyperparameters is specified, and all potential combinations of these hyperparameters are tested. The comprehensive method guarantees the identification of the optimal collection of hyperparameters, but it can take a while, especially for large datasets and intricate models. In a random search, hyperparameters are selected at random from a distribution, and the model’s performance is assessed for each set of hyperparameters. For large datasets and complex models, this strategy can prove more effective and is often less computationally expensive compared to grid search.
• Sequential model-based optimization: Bayesian optimization, which is additionally known as sequential model-based optimization (SMBO), provides a strong and effective strategy for improving functions that are difficult to assess and have intrinsic complexity. In the area of HPO for machine learning models, it is particularly well-liked. To find the best solution, SMBO iteratively explores the search space using a probabilistic representation of the goal function. Gaussian processes or tree-structured parzen estimators are the most often employed surrogate models. The objective function’s uncertainty and behavior are estimated by the surrogate model. When determining the optimal configuration of hyperparameters for machine learning models is time- or money-consuming, Bayesian optimization is a sophisticated optimization technique that is frequently used. The underlying idea is to create a statistical surrogate approach, frequently a Gaussian method, to approximate a true function. To strike a balance between exploring uncharted territory and taking advantage of promising ones, our surrogate model directs the search by making recommendations for which hyperparameters to test next. Throughout this procedure, the representative model is updated, the obtaining function is optimized, the true objective is assessed, and new observations are added in an iterative loop. Bayesian optimization is renowned for its effectiveness, capacity to handle high-dimensional and complex search spaces, and ability to adapt to noisy functions. Bayesian optimization methods are capable of optimizing continuous hyperparameters; categorical hyperparameters cannot be improved directly. In Bayesian optimization, a particular approach is applied called the Tree of Parzen Estimators (TPE). It is a way to model the distribution of the objective function and to decide where to sample next after modeling the distribution. TPE has a reputation for effectiveness and efficiency in directing optimization. For every hyperparameter, TPE keeps two probability density functions (PDFs): one for the optimal configurations that have produced improved outcomes and another for the undesirable configurations. Modeling the distribution of hyperparameters is done using these PDFs. TPE balances exploitation (sampling areas with potential high values) and exploration (sampling areas with potential high values) by taking into account the ratio of PDF values in the favorable and unfavorable configurations. The Tree of Parzen Estimators method handles categorical hyper-parameters in a tree-structured fashion. Contrary to Bayesian, the Parzen Estimators Tree technique handles categorical hyperparameters in a tree-structured fashion. TPE and Bayesian optimization are both effective methods for tuning and optimizing hyperparameters. The effectiveness of TPE, in particular, in directing the search procedure based on prior evaluations is well acknowledged.
• Genetic algorithms and evolutionary strategies: The principles of natural selection and evolution served as inspiration for genetic algorithms and evolutionary methods, two types of optimization approaches. Both methods have proven successful in generating suitable hyperparameter settings for various machine learning tasks, particularly in scenarios involving extensive and intricate search spaces or where the target function is uncertain or computationally costly to assess. Both systems follow the same process, which involves maintaining a population of feasible options and improving this population continuously to generate better answers over time. While evolutionary procedures are better suited for continuous parameter spaces, genetic algorithms concentrate on discrete parameter spaces. Numerous optimization problems, such as hyperparameter tuning, neural architecture search, and engineering design, use both techniques.
• Optimization libraries: Specialized optimization libraries like Optuna, Hyperopt (HO), and Ray Tune are frequently included in AutoML platforms. The AutoML pipeline may be readily combined with these libraries, which provide a variety of search algorithms. First up is Ray Tune, a dedicated library for hyperparameter tuning that facilitates the fine-tuning of machine learning frameworks like PyTorch, XGBoost, Scikit-Learn, TensorFlow, and Keras. Leveraging advanced techniques such as Population-Based Training (PBT) and HyperBand/ASHA. Moreover, it integrates seamlessly with a plethora of other HPO programs, including Ax, BayesOpt, BOHB, Dragonfly, FLAML, HEBO, Hyperopt, Nevergrad, Optuna, SigOpt, skopt, and ZOOpt. Another is Optuna, which primarily uses Bayesian optimization, a probabilistic model-based optimization technique, when searching for ideal hyperparameters. It simulates the behavior of the objective function and decides which hyperparameters to investigate next based on the model. Ray Tune offers several techniques for generating hyperparameter suggestions, including TPE and CMA-ES (Covariance Matrix Adaptation Evolution Strategy). Additionally, it employs pruning techniques, also known as automatic early-stopping, such as Asynchronous Successive Halving and Median Stopping, to terminate the evaluation of unfavorable configurations early, thereby conserving computational resources. The last one, Hyperopt, uses Bayesian optimization techniques to find the best hyperparameters. The optimization algorithms TPE and adaptive TPE are used by Hyperopt to swiftly discover the best hyperparameters.
• Hyperband and successive halving: Two methods created for effective HPO in machine learning, Hyperband and successive halving, are closely linked. These techniques are founded on the idea of early pausing and iteratively eliminating underperforming configurations. The theory states that configurations that perform well after fewer iterations are more likely to be promising and can perform even better with additional resources. Successive halving effectively eliminates subpar configurations and concentrates resources on those with the potential to produce higher results. They work especially well when working with constrained computational resources.
• Neural architecture search (NAS) and gradient-based optimization: Within the domain of deep learning, NAS and gradient-based optimization represent two indispensable paradigms, each bearing distinct yet intricately interconnected roles in the multifaceted realms of model development and optimization. NAS, an eminent technique, assumes the pivotal task of automating the architectural design of neural networks. This endeavor aspires to discern an optimal network configuration tailored to a specific task, thereby potentially ameliorating performance benchmarks and mitigating human exertion. A preset search space of potential architectures is analyzed by NAS algorithms to find configurations that produce high-performing models. Gradient-based optimization, a venerable class of optimization methodologies, hinges upon the utilization of the gradient (or derivative) of an objective function. It orchestrates a process of iterative updates to a solution in a bid to ascertain its optima, thereby epitomizing a cornerstone in the pursuit of model refinement and convergence towards superior performance. NAS automates the process of designing neural network architectures, while gradient-based optimization fine-tunes and optimizes various aspects of these architectures. Both concepts contribute to the advancement of deep learning by reducing the manual effort required to design high-performing models and improving the efficiency of model development and optimization.
• Ensemble methods: Ensemble method optimization encompasses the intricate pursuit of identifying the optimal amalgamation of constituent models within an ensemble framework, with the overarching objective of attaining peak predictive performance. Two widely acclaimed ensemble techniques in the realm of machine learning, namely voting and stacking, assume prominence due to their efficacy in consolidating the predictions derived from multiple base models, thereby engendering a final model characterized by heightened accuracy and enhanced resilience. These techniques enhance overall performance and generalization by utilizing the variety and complementary qualities of individual models. Voting is an easy-to-use ensemble strategy that effectively integrates predictions from various models to produce a single final forecast. Both classification and regression problems respond favorably to it. On the other hand, stacking adopts a more intricate approach by learning a meta-model that intelligently combines the predictions provided by various base models. These base models’ predictions assume the role of input features for the meta-model, with stacking’s primary intent being the exploitation of the individual models’ distinctive competencies, culminating in the acquisition of insights into the optimal manner in which their outputs can be harmoniously combined.
• Meta-learning: Meta-learning, often colloquially referred to as ‘learning to learn,’ constitutes a specialized and strategic facet within the domain of machine learning. This approach is uniquely oriented toward the training of models with the inherent capacity to rapidly adapt to novel tasks, exhibiting proficiency even in the presence of limited training data. Meta-learning’s underlying framework is predicated upon the acquisition of knowledge from the outcomes produced by other machine learning algorithms that have undergone the process of learning through exposure to data. Consequently, meta-learning mandates the coexistence of pre-trained learning algorithms as a prerequisite. The overarching aim of meta-learning resides in the cultivation of models characterized by the ability to generalize competently across a diverse spectrum of tasks, leveraging insights and abstractions gleaned from prior learning experiences. This methodology assumes paramount significance when confronted with scenarios wherein the availability of labeled data for individual tasks remains constrained, rendering it an invaluable tool in such contexts.
• Multi-fidelity optimization: A pivotal determinant when configuring multi-fidelity optimization resides in the judicious selection of the budgetary framework employed for the creation of surrogate tasks. Escalating data dimensions and the escalating intricacy of models have compounded the challenge of devising viable configurations within a constrained computational or temporal budget. The fundamental idea behind multi-fidelity optimization is to balance exploration and exploitation by using lower-fidelity evaluations to quickly explore the search space and identify promising regions, and then allocating higher-fidelity evaluations to refine the optimization within those regions. This approach assumes particular salience in scenarios characterized by the exigency of resource conservation, such as those where high-fidelity evaluations entail substantial time or cost investments. Multi-fidelity optimization finds notable applicability in domains encompassing hyperparameter tuning, architectural exploration, and model selection.

AutoML has primarily found application within the domain of supervised learning, encompassing regression and classification tasks; however, its versatility extends to unsupervised learning and natural language processing, as briefly outlined in Table 2. In essence, AutoML epitomizes the automation of the entire spectrum of machine learning processes, commencing with data pre-processing and culminating in model construction, all undertaken with the overarching objective of optimizing performance on extensive datasets. AutoML inherently encompasses the automated orchestration of critical facets such as model algorithm selection, HPO, iterative modeling refinement, and rigorous model evaluation. The goal is to simplify machine learning efforts, greatly reducing manual coding and eliminating the need for laborious hyperparameter tuning. The crux of AutoML’s innovative framework is fundamentally anchored in hyperparameter search techniques, wielded not only for the optimization of preprocessing components but also for the judicious selection of model types and the fine-tuning of their hyperparameters. Irrespective of the specific approach undertaken, AutoML emerges as a potent instrument for rendering machine learning more accessible and efficient. The AutoML tools enumerated below constitute the focal instruments deployed within the purview of this research.

**Table 2:** Functionality comparison table for AutoML tools. Within the scope of this article, the features of AutoML tools that are considered important are shared.

**Tool**		**FLAML**	**AutoViML**	**EvalML**	**Pycaret**	**AutoGluon**	**H2OAutoML**	**Tpot**
ML Task	Supervised learning	✓	✓	✓	✓	✓	✓	✓
	Unsupervised learning				✓	✓	✓	
	Neural network					✓	✓	
	Natural language processing	✓	✓		✓	✓	✓	
	Time series	✓		✓	✓	✓	✓	
Hyperparameter optimization	Grid search and random search	✓	✓	✓	✓	✓	✓	
	Bayesian search	✓	✓	✓	✓	✓	✓	
	Genetic algorithms and evolutionary strategies							✓
	Hyperband and successive halving				✓			
	Neural architecture search					✓		
	Ensemble method	✓	✓	✓	✓	✓	✓	✓
	Meta-Learning						✓	
	Multi-Fidelity	✓						
Time	Allow maximum limit search time	✓			✓	✓	✓	
	Restrict time	✓		✓	✓	✓	✓	✓
Result analysis and visualization	Model leaderboard	✓	✓	✓	✓	✓	✓	✓
	Feature importance	✓	✓	✓	✓	✓	✓	
	Model explainability		✓	✓	✓		✓	
	Metrics customization	✓		✓	✓	✓	✓	✓

### FLAML

FLAML, developed by Microsoft Research Wang et al. (2021), stands as a streamlined Python library meticulously engineered to harness state-of-the-art algorithms characterized by their resource efficiency and amenability to parallelization. The primary objective of FLAML is the judicious identification of optimal machine learning models, all accomplished within a framework that is both effective and economically prudent. Notably, FLAML leverages the inherent structural characteristics of the search space, effectively executing simultaneous optimization for cost-effectiveness and model performance. FLAML features two pioneering strategies that emanate from the research endeavors at Microsoft:

• Cost-frugal optimization (CFO): CFO is a strategy that works inside the sequential search approach and aims to maximize model performance and resource allocation. As the search progresses, judgments regarding the selection of learning algorithms, hyperparameter configurations, sample sizes, and resampling techniques are iteratively refined. This progression traverses the continuum from cost-efficient trial executions to those of a more resource-intensive nature, ultimately converging towards highly accurate models.
• BlendSearch: BlendSearch coordinates parallelized search processes, intelligently exploring the search space. In order to inform decisions about learner selection, hyperparameter tuning, sample sizes, and resampling strategies, it dynamically takes into account cost considerations and error rates. Contrary to CFO, BlendSearch does not wait until local convergence has been reached before exploring new starting locations. Instead, it quickly seeks low-cost trial spots for feedback and starts the search process from there, much like the CFO.

FLAML offers users the flexibility to swiftly customize the choice of learners, search domains, and optimization criteria, thereby empowering them to tailor the framework to their specific needs and objectives. Subsequent to these adjustments, FLAML can be seamlessly deployed to streamline the machine learning model selection process.

### H2OAutoML

The H2OAutoML tool developed by LeDell & Poirier (2020), AutoML, is a highly scalable AutoML framework that can support supervised and unsupervised algorithms, neural networks, and other types of algorithms. Without requiring the assistance of a person, training and HPO are carried out within H2OAutoML, leading to the careful selection of an ideal model. The framework provides the option to establish constraints in the form of a maximum runtime or a predefined cap on the number of models generated, allowing for a controlled halt in the training process. A distinctive facet of H2OAutoML lies in its seamless integration of model selection and HPO. In H2OAutoML, hyperparameter tuning is accomplished using grid search algorithms that have been specifically designed for base machine learning models, including GLM, DNN, GBM, XGBoost, XRT, and ensemble models. To elevate the predictive prowess of ensemble methods, H2OAutoML ingeniously fuses random grid search methodologies with stacked ensemble strategies. The training procedure in H2OAutoML encompasses both base and ensemble models, with users retaining the prerogative to prescribe the sequence of training algorithms, contingent upon their respective performances.

The meta-learning algorithm, or meta-learner, is the key component guiding the ideal fusion of these base learners. Surprisingly, the H2OAutoML framework makes it possible to train two different types of stacked ensembles: one kind only uses the best models from each methodological family, while the other type integrates all trained models into its ensemble structure. Furthermore, the H2OAutoML tool extends its functionality to encompass comprehensive, interpretable analyses, aligning seamlessly with the predictions emanating from the premier model.

### TPOT

The primary objective of employing tree-based genetic code aims to methodically investigate a range of streams that comprise different operators intended for uses including feature creation, feature selection, and model analysis. Challenges involving regression or classification are especially well-suited for this paradigm. This approach was used in a work by Olson et al. (2016), and TPOT integrates several machine learning methods, such as aggregate and single tree-based designs, as well as uncertain and probabilistic linear algorithms, among others. Notably, each machine learning algorithm within this library corresponds to a distinct machine learning pipeline operator within TPOT. These pre-established pipelines, in essence, manifest as hierarchical tree structures.

The pivotal mechanism underpinning TPOT entails the iterative crossover or combination of the most proficient predictions, guided by considerations of accuracy and performance at each generational iteration. At the same time, TPOT introduces stochastic modifications to pipeline topologies that are intended to find algorithms with the best performance traits. As a result, models are configured in ways that maximize performance while minimizing complexity. The ‘optimal’ candidate pipeline, as determined by TPOT, manifests as a fusion of models and preprocessing methods painstakingly adapted to the issue area under consideration.

TPOT systematically refines the population for the succeeding generation at the conclusion of each generational cycle by pruning the pipelines demonstrating substandard performance. Through meticulous iteration of these operations, TPOT skillfully establishes a compromise between high performance and model simplicity. This is done in order to achieve an optimum pipeline configuration. Finally, TPOT provides the Python code for the machine learning pipeline with the highest performance, encompassing the entire iterative, performance-driven process.

### AutoGluon tabular

AutoGluon-Tabular (AGT), a cutting-edge open-source AutoML framework developed by Erickson et al. (2020), offers an exceptional ability to automatically determine the data type for each column in accordance with Amazon Web Services (AWS) norms. Furthermore, it exhibits an aptitude for discerning the modeling task at hand, encompassing facets such as regression, multiclass classification, or binary classification, all contingent upon the information encapsulated within the designated label column. With AGT, users may quickly begin training a model with unprocessed information; existing data insights or expertise in machine learning models are not necessary. A single line of Python code is all that is needed to accomplish this simplified procedure. This streamlined process concludes with the training of ensemble algorithms. This integration leads to superior outcomes across a variety of ML tasks. AGT’s robustness is particularly highlighted by its ability to continue training even in situations where individual machine learning models fail.

For model training and validation, AutoGluon, which is well-known for its ensemble model creation methodology, uses a technique where the dataset is automatically partitioned among many folds. A diverse set of models is fitted, encompassing k-Nearest Neighbors, random forests, CatBoost-boosted trees, extremely randomized trees, neural networks, and user-specified models. The performance standards of any solitary trained model are then exceeded by an optimized model ensemble. A multi-layer stacking design with a base layer and a minimum of one stacked layer is also part of the AutoGluon approach. A range of base models are included in the fundamental layer, and the forecasting results from these models are aggregated and used as inputs for stacker models that are part of the next layer. Greater stacking layers use these stacker models as their basic models. Interestingly, AutoGluon uses the same hyperparameter values for stackers in every layer, following a consistent model selection process for stackers. This method aids in lowering the computational burden related to the optimization of hyperparameters and the algorithmic selection process.

Moreover, AutoGluon enhances stacking modeling in higher layers by making them more flexible and enabling the use of both the original input characteristics and training predictions from layers before. A salient feature entails the employment of ensemble selection techniques, whereby the final stacking layer adeptly weighs and amalgamates the predictions furnished by the stacker models. Users retain control over hyperparameter operations within AutoGluon, with access to pre-defined settings categorized as ‘best quality’, ‘high quality’, ‘good quality’, and ‘medium quality’. It is imperative to note that there exists a trade-off between accuracy, performance, and inference speed as one transitions away from the ‘best quality’ settings.

### AutoViML

The Automatic Variant Interpretable Machine Learning Model, which is built on Python, is a flexible framework that is primarily designed for use with huge datasets while emphasizing interpretability. Central to its functionality is the automated harnessing of multiple variables from the dataset in conjunction with an ensemble of diverse machine learning models. Of particular note is the way that AutoViML smoothly coordinates the process of choosing features, which yields a low-commitment yet highly effective model with a reduced feature set.

Two pivotal algorithms underpin AutoViML’s feature selection prowess:

• SULOV algorithm (Searching for Uncorrelated List of Variables): As the acronym implies, the SULOV method embarks on the quest for an uncorrelated array of variables. In essence, this technique entails the curation of features that exhibit a high degree of association with the classification variable while simultaneously ensuring their mutual dissimilarity and absence of correlation.
• Recursive XGBoost approach: Building upon the SULOV methodology, the recursive XGBoost method takes center stage. It endeavors to discern the optimal variables by weighing the XGBoost feature importance metric against successively diminishing subsets of the dataset, thereby iteratively refining the variable selection process.

Datasets are accepted by AutoViML in the form of Pandas dataframes, demonstrating its outstanding flexibility in handling them. It skillfully handles a wide range of variable kinds, including text, date/time, structural (such as lists and dictionaries), numeric, boolean, factor, and categorical variables, all within a single modeling paradigm, made possible by a streamlined process. Users are relieved of the burden of prepping the dataset since AutoViML performs data scrubbing, variable categorization, feature reduction, and model training deftly and effortlessly. Furthermore, the system uses a trinity of automated HPO approaches, including grid search, randomized search, and Hyperopt, to efficiently and precisely set the model’s parameters.

Beyond its prowess in model development, the AutoViML library emerges as a valuable asset in the realm of model interpretation and analysis. It serves as a potent tool for elucidating the intricacies of black-box algorithms, rendering their operational processes more comprehensible. Furthermore, by championing the creation of parsimonious models comprising the minimal essential feature set, AutoViML fosters interpretability, abetted by the SHAP (Shapley Additive Explanations) library. Additionally, AutoViML extends its utility by offering a repertoire of graphical representations and visualizations, further enhancing our comprehension of model efficacy and behavior.

### EvalML

The Innovation Labs team carefully crafted the EvalML library, which works in perfect harmony with two essential frameworks: Compose, an instrumental framework that specializes in automated prediction engineering, and Featuretools, a framework that is well-known for its skill in automated feature engineering. This holistic amalgamation equips practitioners with a robust arsenal for proficiently navigating the multifaceted landscape of AutoML.

Featuretools emerges as a linchpin in the feature engineering facet, demonstrating unparalleled acumen in the manipulation of temporal and relational datasets. Its prowess is most pronounced in the creation of feature matrices, a critical underpinning of machine learning workflows. Conversely, Compose plays a pivotal role in orchestrating prediction problems and curating labels for supervised learning tasks, endowing users with a versatile toolkit for formulating and structuring predictive challenges.

EvalML, at its core, offers a rich repository of modeling libraries, underpinned by a unified and intuitive API for the streamlined creation of machine learning models. Augmenting its functionality, EvalML introduces an innovative data structure termed ‘DataTable’, which bestows the capability to differentiate columns based on their shared physical data type. This feature-rich data table design provides a unified environment for selecting models, hyperparameter efficiency improvements, and a subtle selection of features.

Feature selection within EvalML leverages the robust Random Forest classifier/regressor, strategically employed to distill pertinent features for model training. EvalML’s workflow is centered around the use of Bayesian optimization as its default optimizer, which directs the process of finding the best training pipeline that meets the specified goal. The architecture of EvalML inherently supports the construction and optimization of machine learning pipelines contingent upon a user-defined objective function parameter. Furthermore, custom-defined objectives prove instrumental in the ranking of models within the AutoML leaderboard, both during and after the search process. This versatility positions custom objectives as invaluable tools for guiding the AutoML search towards models imbued with maximal impact and relevance.

In addition to its prowess in automated pipeline optimization and ensemble stacking, EvalML offers an array of interpretability modules, serving as a beacon of transparency in model analysis. Users are empowered to delve into the intricacies of model predictions through SHAP values and Local Interpretable Model-Agnostic Explanations (LIME). These interpretability techniques provide nuanced insights into the salient characteristics influencing a particular prediction, offering a granular perspective that transcends traditional feature importance assessments. This holistic featuretools/EvalML pipeline not only excels in the realms of explainability and transparency but also provides users with intuitive natural language explanations, further cementing its standing as an indispensable choice in complex machine learning scenarios.

### PyCaret

PyCaret, an open-source library developed by Ali (2020), presents an invaluable resource for streamlined and expeditious experimentation with machine learning models. Its salient feature lies in its capacity to orchestrate machine learning operations, encapsulating them within a well-defined pipeline, thereby facilitating seamless model deployment and subsequent execution. The library boasts an extensive ensemble of machine learning frameworks and libraries, encompassing luminaries such as XGBoost, CatBoost, Hyperopt, LightGBM, and Scikit-Learn, among others. Furthermore, PyCaret houses a comprehensive repository of over 70 models, spanning both supervised and unsupervised domains. Within its ambit, PyCaret accommodates a spectrum of ensemble techniques, including bagging, boosting, and stacking.

Conceptually, PyCaret may be conceptualized as a Python wrapper module thoughtfully equipped with embedded libraries capable of harnessing a multitude of methodologies for executing diverse machine learning tasks. It affords users a holistic ecosystem, encompassing data preprocessing, model training, model interpretability, and exploratory data analysis, bolstered by the integration of the AutoViz package. Addressing a pervasive challenge in machine learning, PyCaret extends support for handling missing data, offering imputation strategies grounded in mean values for categorical features and constant values for numeric features. Additionally, the library offers a gamut of data scaling and transformation options, instrumental in mitigating variance and reshaping distribution profiles.

Augmenting the predictive accuracy of its in-built models, PyCaret incorporates hyperparameter tuning and ensemble methods as integral components of its toolkit. The process of hyperparameter tuning is underpinned by a meticulous selection of the top-performing models, predicated upon prediction scores. PyCaret seamlessly interfaces with a spectrum of hyperparameter tuning algorithms, encompassing Random Search, Grid Search, and Bayesian Search, while harnessing the capabilities of esteemed libraries such as Scikit-learn, Optuna, Tune-Sklearn, and Ray. Interestingly, the library combines the adjusted models using the powerful bagging technique, which is well known for improving the reliability and predictive power of regression models.

PyCaret offers an array of visualization tools, empowering users to scrutinize data, evaluate model performance, and elucidate findings. Furthermore, PyCaret seamlessly integrates with the MLFlow experiment tracking library and the SHAP library, affording users the means to elucidate the outcomes of intricate tree-based machine learning models, thus enhancing model interpretability.

## Dataset

The textile sector, a field known for its prodigious data output, is home to substantial data holdings that can be found there. Contemporary textile manufacturing, characterized by its relentless pursuit of efficiency and precision, has seen a proliferation of electronic devices endowed with high-acquisition-rate sensors. These sensors, now ubiquitous in textile manufacturing, are instrumental in capturing a continuous stream of real-time data, heralding a new era in data-rich operations, according to Lee, Lin & Chang (2018). This technological transformation has culminated in the availability of a diverse and multifaceted data repository, amenable to rigorous statistical modeling, designed to anticipate critical operational parameters within the intricate tapestry of manufacturing processes.

Data collection is carried out in a painstakingly coordinated procedure. The initial step involves accurately inputting necessary data into the ERP system, which serves as the key component of managing information. Then, this data is seamlessly sent to a cloud-based platform, where it is meticulously examined statistically. The fact that this data reservoir was derived directly from the research and development (R&D) company of Iletisim Yazilim’s (1994) ERP system and serves as an accurate representation of actual manufacturing processes highlights its authenticity and applicability.

The dataset under investigation is a composite entity that brings together information about the characteristics of textile machinery and fabric properties. Fabric properties, constituting a vital dimension of the dataset, are culled from three distinct tables sourced from the textile company’s comprehensive database. The first of these tables, encompassing 56,002 records, delineates manufacturing parameters and the causal factors behind production stoppages. The second table, comprising 12,544 records, provides a comprehensive exposition of yarn types and their specific applications. The third table, replete with 124,104 records, delineates quality-related information.

This data gathering project, which took place over the course of two years, produced a database with over 359,237 rows and 52 columns, each representing a unique aspect of the data landscape. The study that follows focuses on the most important data properties and traits, which are described in Table 3. These characteristics cover a wide range of data, including yarn properties, quality indicators, application domains, production process variables, and perceptions of production outages and their durations. The complexity of textile manufacturing processes is notably exacerbated by the intricate interplay of myriad variables, including but not limited to processes, machinery, engine RPM, and constituent components. This intricate web of interactions makes the characterization of operational parameters a formidable challenge. The creation of final fabric products, a culmination of diverse yarn types and manufacturing processes, further accentuates the intricacy of the textile manufacturing landscape.

**Table 3:** Attributes in the dataset and their descriptions.

**Attribute**	**Data Type**	**Description**	
**StockCode**	Object	It is the identification number that the factory defines for the product.
**PatternNo**	Object	Pattern number of the product.
**Construction**	Object	It reveals the fiber type and the product’s proportion.
**Width**	Float64	The width of the fabric as it emerges from the loom.
**FiberType**	Object	Explanation of the abbreviation for use in construction.
**ProductClass**	Object	Class of the manufactured product.
**LotNo**	Object	Number given to yarn combinations.
**LoomNo**	Object	On which loom the fabric is woven.
**FabricRollNo**	Object	The computer system gives each roll of fabric a unique number.
**FilamentsNumber**	Int64	It represents how many fibers it consists of.
**Fabric roll position**	Object	In some cases, the fabric roll synchronously touches the top and bottom of the machine at the same time;
**-**		this parameter shows its instantaneous position.
**EngineRpm**	Float64	It is the number of wefts that the machine makes on average per minute.
**YarnStockNo**	Object	It indicates the type of yarn used to make the fabric.
**YarnUsageArea**	Object	Identifies the location of the yarn on the loom.
**YarnNo**	Float64	It represents the thickness of the yarn, and a smaller value indicates thicker yarn.
**YarnNoTpye**	Object	Yarn count types.
**YarnBlend**	Object	Yarn blends ratio and fiber groups.
**FabricRollMeter**	Float64	Before quality control roll fabric length.
**TwistsNumber**	Int64	It is the process of twisting the fibers that make up the yarns around their own axis with the help of a machine or by hand.

The dataset, an invaluable confluence of historical production process data and experiential insights, is a testament to a data-driven approach. It integrates machine operational parameters culled from various textile operations, including weft, warp, pile, and creel, with experiential data tracking quality control processes and the outcomes of failure assessments. In order to unravel the complex web of textile manufacturing, this multidimensional dataset brings together data science and operational know-how.

### Feature engineering

The dataset is proof that a data-driven strategy works because it brings together historical production process data with firsthand knowledge. It combines operational machine parameters drawn from a range of textile operations, such as weft, warp, pile, and creel, with empirical data tracing quality control procedures and the results of failure assessments. Effectively, it entails the condensation and summarization of the initial set of features into a more compact and manageable form.

The quality assurance protocols germane to loom-produced artifacts constitute a distinct facet of the manufacturing process. These protocols, centered on meticulous quality assessment, are orchestrated manually by a cadre of highly skilled personnel. A salient characteristic of this quality control regimen is its inherent imbalance, epitomized by the pronounced discrepancy in the quantities of items categorized under different quality tiers. To provide contextual clarity, the composition of this quality distribution is elucidated: the inventory comprises a substantial 257,445 first-quality items, accompanied by a markedly diminutive cohort of 11,017 second-quality items, and a scant ensemble of 12 items relegated to the third-quality category.

It is crucial to stress that the complex nature of this quality control procedure makes it impossible to develop an accurate and clear-cut quality estimation characteristic. The inherent imbalance in quality distribution, which makes it impossible to develop a quality metric that can be used everywhere, emphasizes this distinction.

Utilizing the attributes delineated in Table 4, encompassing parameters such as failure quantity, duration, and quality, a novel attribute is derived through the application of the following mathematical equation:

(1) \begin{eqnarray*}SQT=BWeQ+TWeQ+BWaQ+TWaQ+PQ+UQ+CQ\end{eqnarray*}

(2) \begin{eqnarray*}SDT=BWeD+TWeD+BWaD+TWaD+PD+UD+CD.\end{eqnarray*}

**Table 4:** Attributes to use for feature extraction.

**Abbreviation**	**Attribute**	**Data type**	**Description**
**BWeQ**	**BottomWeftStopQuantity**	Float64	The amount of breakage in the shuttle of the bottom fabric roll.
**TWeQ**	**TopWeftStopQuantity**	Float64	The amount of breakage in the shuttle of the upper fabric roll.
**BWaQ**	**BottomWarpStopQuantity**	Float64	The amount of stop in the warp yarn at the bottom of the loom.
**TWaQ**	**TopWarpStopQuantity**	Float64	The amount of stop in the warp yarn at the top of the loom.
**PQ**	**PileStopQuantity**	Float64	The amount of stopping of the pile yarn on the weaving loom.
**UQ**	**UndefinedStopQuantity**	Float64	Problems other than the reasons mentioned.
**CQ**	**CreelStopQuantity**	Float64	Usually used in jacquard machines.
**BWeD**	**BottomWeftStopDuration**	Float64	The duration of breakage in the shuttle of the bottom fabric roll.
**TWeD**	**TopWeftStopDuration**	Float64	The duration of breakage in the shuttle of the upper fabric roll.
**BWaD**	**BottomWarpStopDuration**	Float64	The duration of stop in the warp yarn at the bottom of the loom.
**TWaD**	**TopWarpStopDuration**	Float64	The duration of stop in the warp yarn at the top of the loom.
**PD**	**PileStopDuration**	Float64	The duration of stopping of the pile yarn on the weaving loom.
**UD**	**UndefinedStopDuration**	Float64	Problems other than the reasons mentioned.
**CD**	**CreelStopDuration**	Float64	Usually used in jacquard machines.
**Qua**	**Quality**	Int64	Quality classification of fabrics.
**QCFRM**	**QCFabricRollMeter**	Float64	After quality control roll fabric length.

In Eqs. (1) and (2), total quantity and downtimes for each product were obtained from the machine breakdowns that occurred. Let new quality production parameter (NQPP) represent the new quality production parameter, SDT represent the stop duration total, SQT represent the stop quantity total, QCFRM represent the quality control factor for raw materials, and Qua represent the quality parameter. Then, NQPP can be defined as: Utilizing the attributes delineated in Table 4, encompassing parameters such as failure quantity, duration, and quality, a novel attribute is derived through the application of the following mathematical equation:

(3) \begin{eqnarray*}NQPP= \frac{ \frac{SDT}{SQT} }{QCFRM} \cdot Qua.\end{eqnarray*}

We have computed the downtime per failure occurrence for each product, followed by the normalization of this novel parameter by the fabric meter, thereby yielding the downtime per unit of fabric meter. This newly established attribute has been associated with the unstable quality class, consequently facilitating the creation of an additional attribute. The utilization of the parameter outlined in Eq. (3) permits the concurrent monitoring of both the output quality from the loom and the duration of loom malfunction instances. Through this approach, a comprehensive quality classification system has been formulated, encompassing various influencing factors such as product type and loom performance.

### Data preparation

In certain instances, certain variables in their current form may not be amenable for direct integration into a machine learning model. To make sure the data is suitable for modeling, it is essential to go through a comprehensive data cleaning and preparation procedure before implementing a machine learning model. Pre-processing techniques become indispensable when dealing with data that exhibits issues such as class imbalance, noise, or a significant volume of missing values. As expounded by Kaur, Pannu & Malhi (2019), traditional machine learning approaches prove ineffective when confronted with severely skewed datasets. In our study, we opted to convert all object-type properties into numerical representations upon importing the data from the Excel file using Pandas dataframes. This conversion was necessitated by the presence of numerous data elements in object format, which may not be supported by various AutoML tools. Subsequently, we conducted a thorough examination for NaN or null values within the dataset.

In order to prevent bias during the model training phase, samples that were identical on a row basis were removed from the dataset. During the data preparation step, any missing value data, non-registered material information, and incomplete date entries were meticulously eliminated. Higher than expected RPM values and engine RPM values close to zero in the Engine RPM characteristic were carefully removed from the data set as they indicate that the machine is inadvertently running at idle. Even after these preliminary data cleansing steps, it was observed that the ranges of variables continued to exhibit substantial disparities. Therefore, both continuous and categorical data were subjected to data normalization approaches. Continuous data were initially normalized to standardize the strength of each variable’s impact on the model results. Additionally, for the employed modeling methodologies to be fair, the data attribute ranges must be reasonably consistent with one another; otherwise, some features may unjustly dominate the model. Scaling of feature data, as denoted by Eq. (4), was carried out through normalization, narrowing the data range to a standardized interval, such as 0 to 1 or -1 to 1. Our dataset underwent scaling utilizing the Min-Max normalization algorithm, subsequently falling within the -1 to 1 range. Unfortunately, due to the commercial organization’s reluctance to reveal specific information about the dataset, we are unable to demonstrate the distribution of the original data values.

(4) \begin{eqnarray*}\widetilde {X}= \frac{x-min}{max-min} .\end{eqnarray*}

X represents the input variable. The numbers min and max denote the lowest and highest points in the data, respectively, and $\widetilde {X}$ denotes the normalized value.

Subsequently, it became evident that the dataset encompassed a multitude of categorical variables characterized by a notably extensive cardinality. Conventional techniques for numeric conversion of categorical data, such as one-hot encoding, were applied to variables exhibiting limited cardinality. However, the adoption of one-hot encoding for categorical input variables with exceptionally high cardinality would lead to a substantial expansion of the output space. This transformation would yield a conspicuously sparse representation, potentially hindering the learning process of regression models. Moreover, it would substantially augment the computational memory and processing demands. In order to address this issue, the following study transforms category properties with large variance using the Sin/Cos coding functions Eqs. (5) and (6):

(5) \begin{eqnarray*}{x}_{sin}=sin\ast ( \frac{2\ast \pi \ast X}{H} )\end{eqnarray*}

(6) \begin{eqnarray*}{x}_{cos}=cos\ast ( \frac{2\ast \pi \ast X}{H} ).\end{eqnarray*}

In the context of this study, ‘X’ signifies the input variable under consideration, while ‘H’ represents the total count of categorical variables pertaining to the input attribute. The primary objective involves the creation of two novel features through the application of sine and cosine transformations. Consequently, the original raw column may be dispensed with. An inherent challenge arises from the symmetry of the generated graph around its inflection points. When employing only sine encoding, a situation arises wherein disparate timestamps yield identical sine encodings within a single cycle. To address this limitation and ensure the generation of distinct values within a cyclic context, a complementary cosine transformation, characterized by a phase offset relative to the sine transformation, is introduced. Furthermore, it is noteworthy that the dataset has undergone scaling to normalize its values within the range of [−1, 1]. This standardized scaling has been uniformly applied to both continuous and categorical data attributes, ensuring a consistent scale representation across diverse data types. Notably, empirical research conducted by Mahajan et al. (2021) has established that ordinal encoding may yield suboptimal results in the context of logistic regression and linear regression. Conversely, the adoption of sine and cosine encoding techniques has demonstrated enhanced performance. Furthermore, it is worth noting that the application of one-hot encoding has been found to be counterproductive in the context of classification and regression tree models.

The dataset consisted of 268,474 rows, 25 entries, and one target column following feature engineering and data preprocessing. The entire dataset comprises three subsets: the training dataset, the validation dataset, and the test dataset. Specifically, 80% of the data is allocated for training, 10% for validation, and another 10% for testing purposes.

### AutoML implementation and experimental environment

The computational experiments involving AutoML were scripted in the Python programming language and executed within the Windows operating system environment. Consequently, AutoML models compatible with Linux-based operating systems, such as AutoSklearn, were not included in the study’s methodology. Notably, certain frameworks, including FLAML, are occasionally prone to encountering errors when a solution cannot be ascertained within the initially allocated time frame. This condition is characterized as a ‘bad allocation error’ which, intriguingly, can often be mitigated by marginally extending the time allocation. In the present investigation, this challenge was addressed by incrementally adjusting the time frame from 3600 s to 3700 s. It is interesting to note that several frameworks have a constant propensity to terminate much later than the designated runtime limit. Moreover, it is essential to underscore that while custom metrics were employed to reevaluate AutoML models, an initial objective function was mandated as input. The writer directly chose the objective functions, training durations, and hyperparameter optimization techniques during the experimental setup; these factors were not left up to the AutoML tool’s discretion. It was determined to use MAE as the objective function in our study. Compared to other metrics, MAE is less susceptible to outliers. The hyperparameter configuration in the AutoML tools remained unchanged, and values within their original ranges were utilized, ensuring consistency in our experimental setup. Potential biases introduced by manual adjustment are avoided. Furthermore, flags enabling boosting and stacking algorithms were incorporated into the AutoML frameworks where available. The methodological framework further incorporated a 10-fold external cross-validation strategy, aligning with established AutoML benchmark practices. It was used both to prevent biases in the distribution of data during the training and testing phases and to strengthen the result’s accuracy. The values are averaged, and then the external 10-fold results are determined.

The implementation of both pre-processing and network development tasks utilized Python version 3.7.12 as the programming language. To facilitate the creation and execution of the proposed AutoML models, several specialized frameworks were harnessed, including TPOT (version 0.11.7), H2OAutoML (version 3.40.0.4), AutoGluon (version 0.8.0), PyCaret (version 3.0.2), EvalML (version 0.77.0), AutoViML (version 0.1.710), and FLAML (version 1.2.4). The computational infrastructure utilized for model training was based on a computing system featuring an AMD Ryzen 5 4600H processor, supplemented by Radeon Graphics, running at a clock speed of 3.00 GHz, and equipped with 8.0GB of RAM memory. Notably, it is imperative to highlight that GPU acceleration was intentionally excluded from the computational setup.

### Evaluation metrics

The evaluation of the proposed model’s predictive performance encompassed the utilization of four distinct metrics: the MAE, Explained Variance Score, mean squared error (MSE), and MAPE. The average absolute disparity among the observed values and the related predictions is represented by the MAE, which is computed over the whole dataset. This measure is used to assess the accuracy of predictions. Lower MAE scores signify higher predictive precision. The precise formulations for these error metrics are delineated in Eq. (7)

(7) \begin{eqnarray*}MAE= \frac{1}{N} \ast \sum _{L=1}^{N}\mid {y}_{a}-{y}_{p}\mid .\end{eqnarray*}

The values used in Eqs. (7), (8), (9) and (10) do the following: The three values are *y*_*a*_, *y*_*p*_ and N respectively for the actual, predicted and observational values.

(8) \begin{eqnarray*}MSE= \frac{1}{N} \ast \sum _{L=1}^{N}({y}_{a}-{y}_{p})^{2}.\end{eqnarray*}

The coefficient of determination, denoted as *R*^2^ and expressed in Eq. (9), assumes values within the range of 0% and 100%. An *R*^2^ score of 100% signifies a perfect correlation between two variables, signifying the absence of any variance. Conversely, a low *R*^2^ score suggests a weak correlation, indicative of the inadequacy of the regression model. The mathematical representation of *R*^2^ in equations involves several variables: ‘N’ represents the total count of predicted values, ‘*y*_*p*_’ denotes the predicted value, ‘*y*_*a*_’ signifies the original actual value, and ‘*y*_*average*_’ represents the mean value of the original data. A score of 0 denotes that the model’s performance is equivalent to that of a basic model that can reliably forecast the data’s mean value, while a value of 1 denotes flawless predictive capacity.

(9) \begin{eqnarray*}{R}^{2}({y}_{a},{y}_{p})=1- \frac{\sum _{L=1}^{N}({y}_{a}-{y}_{p})^{2}}{\sum _{L=1}^{N}({y}_{a}-{y}_{average})^{2}} .\end{eqnarray*}

The MAPE, as defined in Eq. (10), serves as a valuable metric for assessing the accuracy of an estimation method. This metric quantifies the accuracy of predicted values relative to actual values by computing the average of the absolute percentage errors across an entire dataset, effectively measuring the average deviation of predictions from actual values. To compute this measure, the absolute difference is divided by the actual value. Consequently, when real values approach or reach zero, the MAPE score may either lead to a division-by-zero error or yield an exceptionally high value. Consequently, it is advisable to exercise caution when employing MAPE in situations where actual values are in close proximity to zero. To mitigate this issue, we refrained from normalizing our target variable. Additionally, lower MAPE values correspond to enhanced model accuracy.

(10) \begin{eqnarray*}MAPE= \frac{1}{N} \ast \sum \frac{(\mid {y}_{a}-{y}_{p}\mid )}{{y}_{a}} \ast 100.\end{eqnarray*}

## Experimental Results

The error rates associated with the performance metrics of root mean squared error, MAE, *R*^2^, and MAPE for each predicted task, or objective function, are presented in Table 5. The findings are presented as the mean of the evaluation scores across ten external folds. The procedure involves identifying the optimal tools for each specific scenario by initially examining the average predicted score for each machine learning model and subsequently assessing the average computational effort, particularly in terms of training time. A similar approach to this process was used by Ferreira et al. (2021). It is important to note that all available HPO methods within the AutoML models listed in Table 2 were meticulously evaluated. Subsequently, Table 5 showcases the AutoML tool and machine learning model that achieved the highest performance scores. For clarity, the optimal values are highlighted using boldface type.

**Table 5:** Experimental result table.

	**FLAML**	**AutoViML**	**EvalML**	**PyCaret**	**AutoGluon**	**H2OAutoML**	**Tpot**
Time Budget	3700 s	None	3600 s	3600 s	3600 s	3600 s	3600 s
Actual train time	3979.52465 s	3530.659857 s	3694.6032 s	4381.6642 s	**3596.3881 s**	3651.2412 s	3694.395345 s
RMSE	27.3337	35.8568	21.4107	25.2598	**21.129**	27.8925	90.4955
Mae	3.2992	16.8826	**2.8282**	3.0291	3.1194	8.6768	18.9063
R2 Score	0.9398	None	0.9602	0.9485	**0.964**	0.9318	0.2572
Mape Score	1.3118	None	1.172	1.235	**1.0444**	3.0771	1.767
Predict time	19.4076 s	None	0.8516 s	0.5951 s	330.4827 s	18.866 s	**0.0979 s**
Best model	LGBM regressor	XGBoost regressor	XGBoost regressor	DecisionTree regressor	WeightedEnsemble_L3	StackedEnsemble	GradientBoosting regressor
Best HPO algorithm	BlendSearch	HyperOpt	–	Optuna - Tpe	–	–	–

The AutoViML framework operates without any imposed time constraints. In contrast, the PyCaret AutoML tool exhibits a notably brief training duration (approximately 801.3350 s) as it first selects the best model and subsequently performs hyperparameter tuning exclusively on this chosen model. The combined time for hyperparameter tuning and model training in PyCaret is itemized in Table 5. However, this approach of not optimizing other models for time efficiency does prompt inquiries into the performance of these untuned models when subjected to optimization. Optimal hyperparameter algorithms are listed in Table 5. Notably, it is crucial to acknowledge that some AutoML tools do not permit the freedom of hyperparameter selection. Consequently, AutoML tools wherein the framework restricts such selections have not been added to Table 4 for the sake of clarity and completeness. The inclusion of the mentioned hyperparameter algorithms in Table 5 follows a meticulous evaluation process. Specifically, these algorithms are appended to the table subsequent to a comprehensive exploration of all the hyperparameter methods proffered by the AutoML tool in question. The selection criterion for inclusion in Table 5 hinges upon the identification of the hyperparameter method that offers the most superior performance results. Also, Table 5 stands as a repository of vital insights, delineating the intricate trade-offs entailed in the algorithmic designs of AutoML tools.

**Figure 2:**
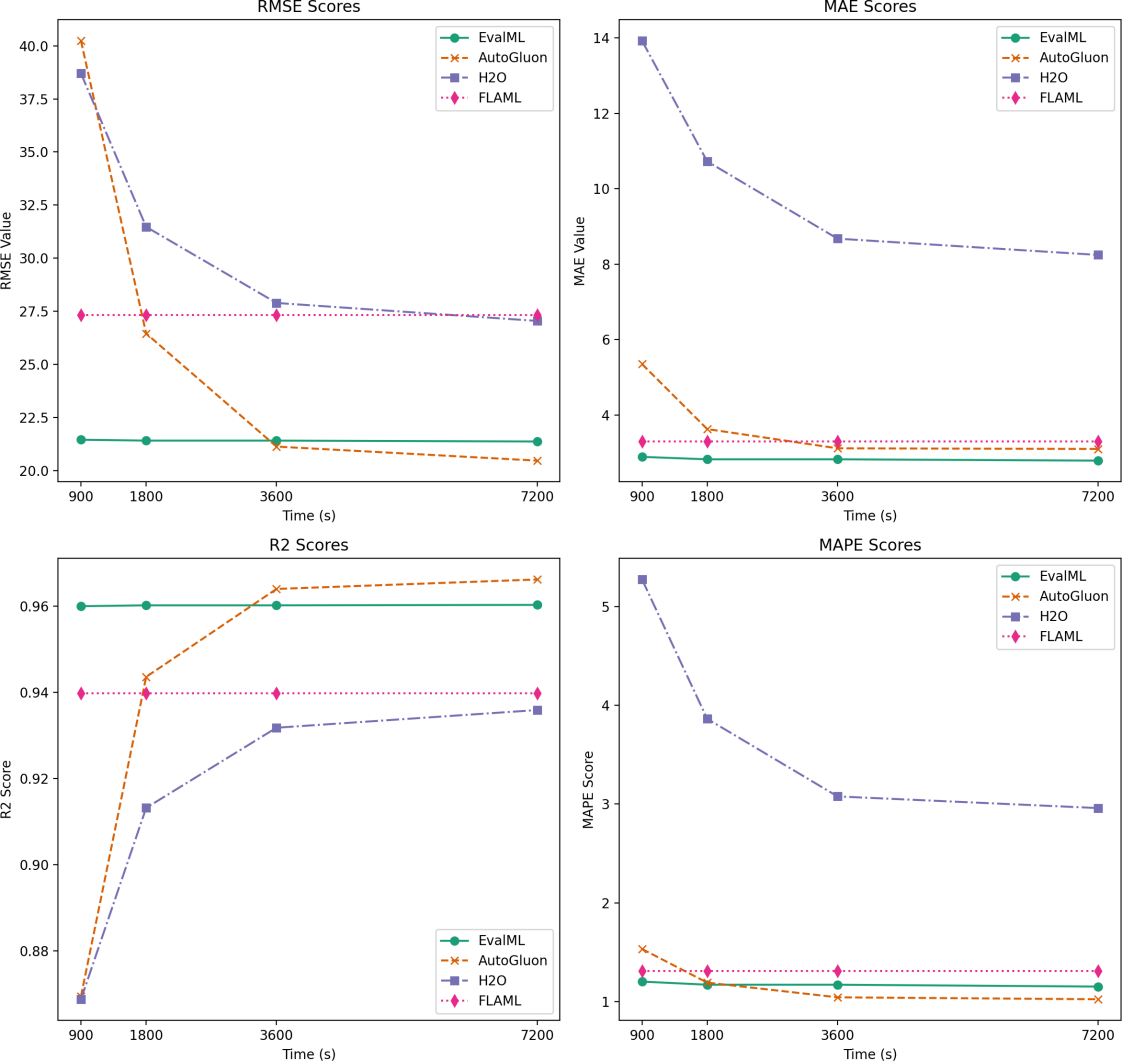
A comprehensive examination at specified time intervals in AutoML metric scores.

PyCaret and AutoViML are excluded from Fig. 2 due to the absence of temporal constraints. Figure 2 is designed to facilitate a comparative analysis of specific metrics, RMSE, MAE, R-squared (R^2^), and MAPE. Additionally, the *X*-axis in Fig. 2 shows how long the training process took in seconds at various intervals of 900, 1800, 3600, and 7200 s. Most of the time, it takes around 900 s for EvalML and FLAML to reach their peak performance among the tools. For the same outcome, H2OAutoML requires a total of two hours. In contrast, after just one hour of training, AutoGluon performs better in practically every measuring statistic. The RMSE is plotted on the *y*-axis in Fig. 2A, where lower RMSE values correspond to better model performance. At short interval times, such as 900s, 1800s, EvalML is shown to have the lowest RMSE score; but, at longer intervals, like 3600s and 7200s, AutoGluon surpasses this performance. However, across all examined times in Fig. 2B, EvalML seems to have the lowest MAE score overall. This demonstrates that, in terms of objective function, it can perform better than the other three AutoML tools. AutoGluon shows an increase in performance over time, as in the RMSE figure. It is evident from Fig. 2C that EvalML obtains a high R-squared score in the time periods of 900s and 1800s. On the other hand, for longer intervals, like 3600s and 7200s, AutoGluon seems to have the greatest R-squared score. Figure 2D indicates that while EvalML clearly outperforms AutoGluon in the first time interval of 900s for MAPE scores, both tools are similar in the subsequent time intervals of 1800s. For longer time intervals of 3600s and 7200s, AutoGluon begins to perform superiorly to EvalML.

The main characteristics, as shown in Table 6, FabricRollMeter, EngineRpm, and LotNo have a higher influence on quality control. The performance of AutoGluon and EvalML is impressive, as shown in Fig. 2. EvalML achieves great efficiency by leveraging features, including PatternNo_cos and PatternNo_sin, that were not previously essential in feature importance, while the AutoGluon AutoML tool is identical to other tools in terms of attribute importance. With the TPOT AutoML tool, there is no feature importance check option available. As a result, it does not include any features for this tool.

**Table 6:** Feature importance result table.

	**FLAML**	**AutoViML**	**EvalML**	**PyCaret**	**AutoGluon**	**H2OAutoML**	**TPOT**
1	FabricRollMeter	EngineRpm	PatternNo_cos	FabricRollMeter	FabricRollMeter	FabricRollMeter	–
2	EngineRpm	FabricRollMeter	PatternNo_sin	EngineRpm	LotNo	EngineRpm	–
3	LotNo	StockCode	EngineRpm	LotNo	EngineRpm	LotNo	–
4	PatternNo_Cos	LotNo	Construction_Cos	PatternNo_cos	LoomNo	PatternNo_Cos	–
5	FabricRollPosition_ OH2	FabricRollPosition_ OH2	FabricRollMeter	FabricRollPosition_ OH2	YarnBlend_cos	FabricRollPosition_ OH2	–
6	LoomNo	PatternNo_Cos	Width	LoomNo	Width	Construction_Sin	–
7	PatternNo_Sin	FiberType_Sin	StockCode	PatternNo_sin	PatternNo_cos	Construction_Cos	–
8	Construction_Cos	YarnStockNo_Cos	LoomNo	StockCode	PatternNo_sin	StockCode	–
9	YarnStockNo_Cos	Width	LotNo	FiberType_Sin	StockCode	LoomNo	–
10	StockCode	LoomNo	FabricRollPosition_ OH2	Construction_Cos	Construction_sin	PatternNo_sin	–

## Result Discussion and Comparison

On the test dataset, the EvalML AutoML model outperforms the other models when compared to the pertinent objective function. EvalML attains the highest performance solely in the context of the mean absolute error objective function. The primary rationale behind this achievement lies in the AutoML tool’s concentrated emphasis on HPO with regard to this specific metric. While we have identified EvalML as the most proficient AutoML tool, it is imperative to scrutinize another notable model, AutoGluon. Diverging from conventional AutoML tools, AutoGluon delivered results effectively within its allocated time budget. Furthermore, AutoGluon exhibited superior performance across the RMSE, *R*^2^, and MAPE performance metrics. A higher *R*^2^ value signifies a more robust connection between the original dataset (the test dataset) and the predicted dataset. This heightened correlation, indicated by the *R*^2^ value, emphasizes the superior performance of the AutoGluon AutoML tool. The MAPE quantifies the average disparity between predictions and actual values. Notably, AutoGluon achieves the lowest MAPE value, reflecting an average absolute percentage difference of 1.044% between its predictions and the actual outcomes. In other words, the model’s predictions deviate, on average, by only 1.044% from the actual results. However, it is worth noting that AutoGluon faces a notable challenge in terms of inference time, specifically the time taken for model predictions. This entails waiting for the results of the selected successful machine learning models and the inference time associated with ensembling these models, as ensemble learning amalgamates decisions from multiple models to enhance overall performance. This invariably impacts inference time. In an automated testing environment, even if accuracy performance wanes, a wait time of 330 s is unlikely to be favored.

The assessment of machine learning model performance is fundamentally contingent on model accuracy; nonetheless, the relentless pursuit of accuracy can precipitate an escalation in model complexity. One pragmatic approach to gauging such complexity entails the measurement of inference speed, typically referred to as ‘predict time.’ In our investigation, we have meticulously quantified ’prediction duration’ while concurrently factoring in performance on the test data to comprehensively evaluate the constraints imposed by models generated through each respective framework. Conversely, we acknowledge that there exist contexts wherein inference time carries nominal significance. However, it is imperative to underscore that, within the purview of our study, prediction time assumes paramount importance. The reason for this is that the new quality score will be predicted, and the loom will be adjusted according to this score. Despite the AutoGluon model garnering one of the highest scores, courtesy of its ensemble approach, it is concurrently associated with the longest prediction time.

Fabric samples are given quality labels based on the subjective assessments of human inspectors; as a result, the dataset produced may have biases or inconsistencies that skew the model’s predictions. In order to obtain a new quality attribute, we thus used the amount of errors, fabric length, and biased quality features through feature extraction. Feature engineering techniques that have been preset are typically used by autoML tools. These algorithms may have inherent biases toward certain data types or problem domains. The final models might be skewed toward numeric characteristics if some pre-processing methods work better on numerical variables and less well on categorical ones. Since AutoML tools such as TPOT do not support categorical coding, the automated pipeline cannot be applied to datasets with categorical features. In order to avoid the biases and errors of AutoML tools, we performed feature engineering and data preprocessing manually and compared the results.

The main reasons why AutoML frameworks perform so well are their built-in machine learning models, large hyperparameter space, and effective techniques for optimization in wide or narrow hyperparameter space. Each AutoML framework analyzes the hyperparameter space and takes a different approach to solving the CASH problem. AutoML tools typically define a search space in which to explore hyperparameter configurations. However, if this search space is too limited, potentially better configurations may be missed. During training, it may prioritize configurations that lead to faster convergence. Therefore, AutoML tools can extend the search space and use different optimization algorithms to reduce the hyperparameter optimization bias. Each AutoML tool compares optimization algorithms internally or with external intervention and is tested for a certain amount of time to search the parameter space. In Fig. 2, the graphical representation unequivocally illustrates that EvalML and FLAML consistently yield commensurate results from the outset. Conversely, AutoGluon and H2OAutoML exhibit a proclivity to progressively approach a more advantageous convergence point over time. The results indicate that the XGBoost method from EvalML performs better in short time intervals and The Weighted Ensemble L3 algorithm from AutoGluon performs better in long time intervals. Users have access to pre-defined settings that are classified and maintain control over hyperparameter operations inside, like AutoGluon. As the “best quality” feature was our choice, we define it as being at the highest point of the hyperparameter space. As a result, AutoGluon provides better performance over long time intervals. The main cause of this phenomena is the HPO process, wherein in certain optimization routines hyperparameter spaces may stagnate after quickly convergent to a local minimum, but in others they may continue until they achieve a global minimum. Both approaches possess their merits; a short-duration, local minimum optimization may be favored when expedited training is of paramount concern, whereas an extended optimization process can be chosen when heightened performance expectations prevail.

Feature importance emerges as a salient facet of machine learning endeavors by affording practitioners the discernment of those attributes within a dataset that wield substantial influence over the ultimate prediction outcome, juxtaposed against features that bear comparatively diminished significance. Despite the appearance of feature harmony in the feature importance rankings of AutoML tools in Table 6, each tool reveals different feature importance hierarchies. Based on information gathered from a competent worker of the firm, FabricRollPosition, Construction, EngineRPM, LoomNo, PatternNo, and FiberType were believed to be the most important aspects determining quality prior to the start of this study. Along with the feature importance table, it now reveals the roll fabric length prior to quality inspection, the number of spins and revolutions that occur on a fixed axis of the looms in a minute, and the numbers assigned to the thread combinations as the three most influential features. Furthermore, feature importance fulfills a pivotal role in enhancing model interpretability within the ambit of machine learning, thereby furnishing insights into the rationale underlying specific model predictions and elucidating strategies for the deliberate manipulation of features to effect alterations in predictive outcomes. Furthermore, this tableau attests to the efficacy of the sin/cos encoding technique, especially when tasked with enhancing the model’s capacity to discern categorical variables characterized by an extensive profusion of distinct, unique values. Sin/cos encoding acquires paramount significance in such scenarios by enabling a more nuanced characterization of the categorical variable, thereby invoking the utilization of two distinct yet congruent attributes to encapsulate the same categorical content. Upon scrutinizing the AutoML tools showcased in Table 6, it becomes conspicuous that a substantial proportion of the sine and cosine features associated with the attribute attain prominent ranks in terms of feature importance.

In textile manufacturing, the production process heavily relies on natural materials such as raw materials, yarn, and machine settings, each varying in quality and properties. Incorporating this variability into AutoML models, especially as new products are introduced, poses significant challenges requiring robust feature engineering and data preprocessing techniques. Ensuring compatibility with legacy systems while minimizing disruptions to existing operations further complicates deployment efforts. Additionally, maintaining data quality and availability for training AutoML models is a persistent challenge, particularly when faced with noisy or incomplete data.

Manual inspection and quality control processes in fabric production are time-consuming and laborintensive. Human errors in manual inspection lead to costly errors and rework. By automating these processes with AutoML, companies significantly reduce labor costs associated with hiring and training personnel for inspection tasks. In addition, AutoML reveals patterns not immediately visible to operators and hidden correlations between production parameters and fabric quality. AutoML can detect potential quality issues early in the production process. It can detect quality problems not only at the level of finished fabric rolls but also at various stages of the production process. By anticipating potential quality issues, manufacturers can implement preventative measures and process optimizations to minimize the likelihood of defects and maintain consistent quality standards. Early detection of quality outcomes results in significant cost savings by reducing material waste and rework. It detects deviations in fabric quality by analyzing production data resulting from factors such as loom features, pattern complexities, fabric differences, and engine power. It allows them to implement preventive measures and contingency plans to minimize the impact on quality standards. This results in higher-quality products that meet customer expectations and reduce the need for rework or returns.

## Conclusions

The idea of Industry 4.0 offers the chance to automatically gather ERP data as well as data from IoT sensors connected to industrial equipment. ML approaches can then be used to shorten lead times and increase productivity. Considering the latest state of technology today, it is a fact that the quality control process in the textile industry will become increasingly automated since machine learning can be applied to nearly any business. Formulating prediction issues, however, presents significant difficulties. To fully understand the context of the business challenge, the data scientist has to collaborate in tandem with domain specialists. Indeed, in reaction to this challenge, numerous open-source AutoML tools have arisen, enabling individuals with limited expertise to develop meaningful machine learning models.

This article provides a comprehensive discussion of the typical AutoML pipeline procedures, encompassing activities such as data preprocessing, feature engineering, automated model selection, and HPO. It also introduces existing tools and libraries for implementing AutoML. While these tools excel in algorithm selection, training, HPO, and benchmarking, they are somewhat limited in their support for labor-intensive tasks like data comprehension, transformation, filtering, preprocessing, and feature engineering. Notably, these benchmarked solutions perform exceptionally well when the input data is clean and of high quality.

This research employs machine learning tools for the training and evaluation of a regression task aimed at early-predicting fabric production quality, leveraging the capabilities of AutoML. To streamline the ML modeling endeavor, an array of AutoML tools, including FLAML, AutoViML, EvalML, AutoGluon, H2OAutoML, PyCaret, and TPOT, were methodically scrutinized. The benchmarking process encompassed an evaluation of computational effort and predictive performance. The methodology for tool selection adopted a multi-faceted approach, wherein the best-performing tools were identified based on both the average predictive scores and the average computational effort expended for each specific task and scenario. For the selected objective function, EvalML offered the best average result among AutoML tools. The actual measurement values and the predicted values were found to have a strong correlation. In real-world scenarios, the time it takes to make predictions (inference time) is a crucial factor. We conducted a comprehensive analysis of the trade-off between inference time and accuracy, uncovering notable disparities in the inference durations among the generated models. The most accurate frameworks achieve superior model precision, but at the expense of slower inference speeds.

As a consequence of these processes, the models obtained are often challenging for humans to comprehend, earning them the characterization of “black boxes.” Enhancing model interpretability, therefore, assumes paramount significance in enhancing the acceptability of AutoML outcomes among domain users. Within the ambit of this study, feature importance rankings were elucidated and subjected to comparison. Additionally, factors exerting an influence on quality estimation were shared and discussed in consultation with domain experts. In future studies, a recommendation system will be developed using the extracted domain information. This envisages a more efficient handling of yarn and fabric patterns, specifying the appropriate looms and engine rpm. With the intent of leveraging more open-source AutoML technology in the future, our goal involves expanding the dataset size. This expansion is particularly aimed at analyzing big data, where the application of deep learning has the potential to yield improved predictions. In the future, as interpretable ensemble models for fabric quality prediction, AutoML techniques can leverage additive models like generalized additive models (GAMs). Because additive models divide the predicted result into additive components that correlate with particular fabric properties, it becomes easier to comprehend how each feature contributes to the overall prediction. In the future, AutoML tools should include well-known techniques that synthetically generate a sample for the minority class in imbalanced data sets, such as Adaptive Synthetic Sampling (ADASYN) and Synthetic Minority Oversampling Technique (SMOTE). In the context of AutoML, it ought to be feasible to use it as an integrated preprocessing stage in pipelines and carry out hyperparameter optimization in these algorithms. As a result, managing class imbalances and future hyperparameter tuning will be automated.

## Supplemental Information

10.7717/peerj-cs.2188/supp-1 Supplemental Information 1 Automated machine learning for fabric quality analysis code and data The raw data is a composite entity that brings together information about the characteristics of textile machinery and fabric properties. Fabric properties, constituting a vital dimension of the dataset, are culled from three distinct tables sourced from the textile company’s comprehensive database. The first table encompassing 56,002 records, delineates manufacturing parameters and the causal factors behind production stoppages. The second table comprising 12,544 records, provides a comprehensive exposition of yarn types and their specific applications. The third table replete with 124,104 records, delineates quality-related information.

## Additional Information and Declarations

### Data Availability

The following information was supplied regarding data availability: The raw data is available in the Supplemental File and GitHub: https://github.com/ttbilgin/FabricDataset.
